# Quantitative proteomics analysis based on data-independent acquisition reveals the effect of Shenling Baizhu powder (SLP) on protein expression in MAFLD rat liver tissue

**DOI:** 10.1186/s12014-023-09442-9

**Published:** 2023-12-01

**Authors:** Sufei Song, Jixian Zheng, Dongmei Zhao, Anni Zheng, Ye Zhu, Qiuling Xu, Tao Liu

**Affiliations:** 1 The First Affiliated Hospital of Hainan Medical University, Haikou, 570102 China; 2 Hainan Medical University, Haikou, 571199 China

**Keywords:** Metabolic associated fatty liver disease, Shenling Baizhu Powder, DIA proteomics, Traditional Chinese medicine

## Abstract

**Background:**

Metabolic associated fatty liver disease (MAFLD) has become the most common chronic liver disease worldwide, and it is also a high-risk factor for the development of other metabolic diseases. Shenling Baizhu powder (SLP) is a traditional Chinese herbal formula with good clinical efficacy against MAFLD. However, its molecular mechanism for the treatment of MAFLD is still not fully understood. This study used quantitative proteomics analysis to reveal the SLP action mechanism in the treatment of MAFLD by discovering the effect of SLP on protein expression in the liver tissue of MAFLD rats.

**Materials and methods:**

Q-Orbitrap LC–MS/MS was used to identify the incoming blood compounds of SLP. The 18 SD male rats were randomly divided into 3 groups (n = 6): control group, HFD group and SLP group. The HFD group and SLP group were established as MAFLD rat models by feeding them a high-fat diet for 4 weeks. Afterwards, the SLP group was treated with SLP (10.89 g/kg/d) for 3 weeks. Biochemical parameters and liver pathological status were measured. Rat liver tissue was analyzed using DIA-based quantitative proteomics and the DEPs were validated by western blotting analysis.

**Results:**

A total of 18 active compounds of SLP were identified and isolated to enter the bloodstream. Comparison of DEPs between control group vs. HFD group and HFD group vs. SLP group revealed that SLP restored the expression of 113 DEPs. SLP catalyzes oxidoreductase activity and binding activity on mitochondria and endoplasmic reticulum to promote lipid oxidative catabolism, maintain oxoacid metabolic homeostasis in vivo and mitigate oxidative stress-induced hepatocyte injury. And 52 signaling pathways including PPAR signaling, arachidonic acid metabolism and glycine, serine and threonine metabolism were enriched by KEGG. PPI topology analysis showed that Cyp4a2, Agxt2, Fabp1, Pck1, Acsm3, Aldh1a1, Got1 and Hmgcs2 were the core DEPs. The western blotting analysis verified that SLP was able to reverse the increase in Fabp1 and Hmgcs2 and the decrease in Pck1 induced by HFD, and the results were consistent proteomic data.

**Conclusion:**

SLP ameliorates hepatic steatosis to exert therapeutic effects on MAFLD by inhibiting the expression of lipid synthesis genes and inhibiting lipid peroxidation in mitochondria. This study provides a new idea and basis for the study of SLP in the treatment of MAFLD and provides an experimental basis for the clinical application of SLP.

**Supplementary Information:**

The online version contains supplementary material available at 10.1186/s12014-023-09442-9.

## Background

In 2020, nonalcoholic associated fatty liver disease (NAFLD) was officially renamed metabolic associated fatty liver disease (MAFLD) [1], which means that the diagnosis of MAFLD emphasizes a focus on metabolic dysfunction rather than the exclusion of other hepatic damaging factors [1, 2]. The pathogenesis of MAFLD is currently unclear and it is generally accepted that it is closely related to factors such as insulin resistance, lipid overload, lipotoxicity and inflammatory processes [3]. Recent studies have shown that MAFLD has a global prevalence of nearly 26% and has become the most common chronic liver disease [4]. In particular, the prevalence of MAFLD in the obese population has exceeded 50% [5], and trending towards younger age groups [6], which undoubtedly places a huge burden on people's health and society [7].

Shenling Baizhu powder (SLP) is a classical formula recorded in the Song Dynasty’s “Taiping Huimin Hejiju Prescription”. It consists of 12 herbs and is commonly used to treat diseases caused by weakness of the spleen and stomach. As traditional Chinese medicine (TCM) believes, “Seeing the disease of the liver, knowing that the liver transmits to the spleen, it is advisable to first strengthen the spleen”, and “spleen qi disperses essence”. The spleen is as transporting center for nutrients, and the “spleen qi” is the driving force for transporting nutrients from the spleen to the whole body. When the "spleen qi" is weak, nutrients cannot be transported throughout the body properly and remain in the liver, leading to liver overload and metabolic imbalance, causing MAFLD development [8–10]. Clinical treatment with SLP can significantly improve the symptoms of MAFLD patients [11, 12].

The treatment of MAFLD in TCM is characterized by a complex mechanism of multiple components, pathways, and targets [13]. Proteomics is the study of the structure and function of the entire protein expressed in the genome of a cell or tissue at a specific time or environment from a holistic level [14]. This dynamic relationship and systematic nature are consistent with the discriminatory and holistic view emphasized in TCM [15] and provides a new way to achieve objectification in TCM. Therefore, we decided to investigate the mechanism of action of SLP in the treatment of MAFLD from a holistic perspective through proteomics.

## Materials and methods

### Experimental drug solutions preparation

SLP was purchased from Guangdong Yifang Pharmaceutical Co. (Guangdong, CN). It consists of 12 herbs including Ren Shen (*panax ginseng*), Bai Zhu (*atractylodes macrocephala*), Fu Ling (*poria cocos*), Yiyi Ren (*coix lacryma*), Sha Ren (*amomum villosum lour*), Lian Zi (*nelumbo nucifera gaertn*), Jie Geng (*platycodon grandifloras*), Baibian Dou (*dolicho lablabl*), Chen Pi (*citri reticulatae pericarpium*), Shan Yao (*dioscoreae rhizoma*), Da Zao (*ziziphus jujuba mill*) and Gan Cao (*glycyrrhiza uralensis*). SLP was dissolved in distilled water at 40 ℃ and configured into 1.89 g drug product/ml of SLP solution, which was stored at 4 ℃.

### Establishment of MAFLD rat model

Sprague–Dawley male rats (6 weeks old, weight 250 ± 20 g) were purchased from Changsha Tianqin Biotechnology Co., td (Changsha, CN), license no. SCXK (Xiang) 2019-0013. All rats were fed in the laboratory animal room of Hainan Medical University, with the room temperature maintained at 22 °C and humidity at 50%. Rats were adaptively fed and watered ad libitum for 1 week, before being randomly divided into control group (n = 6) and MAFLD group (n = 12). The MAFLD rat was established according to a previous study [16]. Normal diet was administered to control group rats and MAFLD group rats were fed a high-fat diet (HFD, 20% lard, 4% sugar, 2% milk powder, 1% cholesterol, 73% standard feed, Suzhou Shuangli Animal Food Company, Suzhou, CN) for 4 weeks. The MAFLD rat model was successfully established by testing liver lipid levels and pathological status.

### Treatment of MAFLD rats

Diets of all the rats continue to be kept the same. Rats in the MAFLD group were randomly divided into the HFD group (n = 6) and the SLP group (n = 6). The human clinical dose (Drug1) was converted to the equivalent dose administered to rats (Drug2) according to the formula: Drug2 (g/kg) = Drug1g/70 kg × 6.3 [17]. The adult clinical dose of SLP was 121 g/d, which was converted to the rat equivalent dose of Drug2 = 121 g/70 kg × 6.3 = 10.89 g/kg, SLP was dissolved in distilled water. Rats in the SLP group were given SLP solution(10 ml/kg/d) by gavage for 3 weeks, and rats in the control and HFD groups were given saline(10 ml/kg/d) by gavage for 3 weeks, after which the blood and liver tissues were collected from the rats for subsequent testing.

**Fig. 1:**
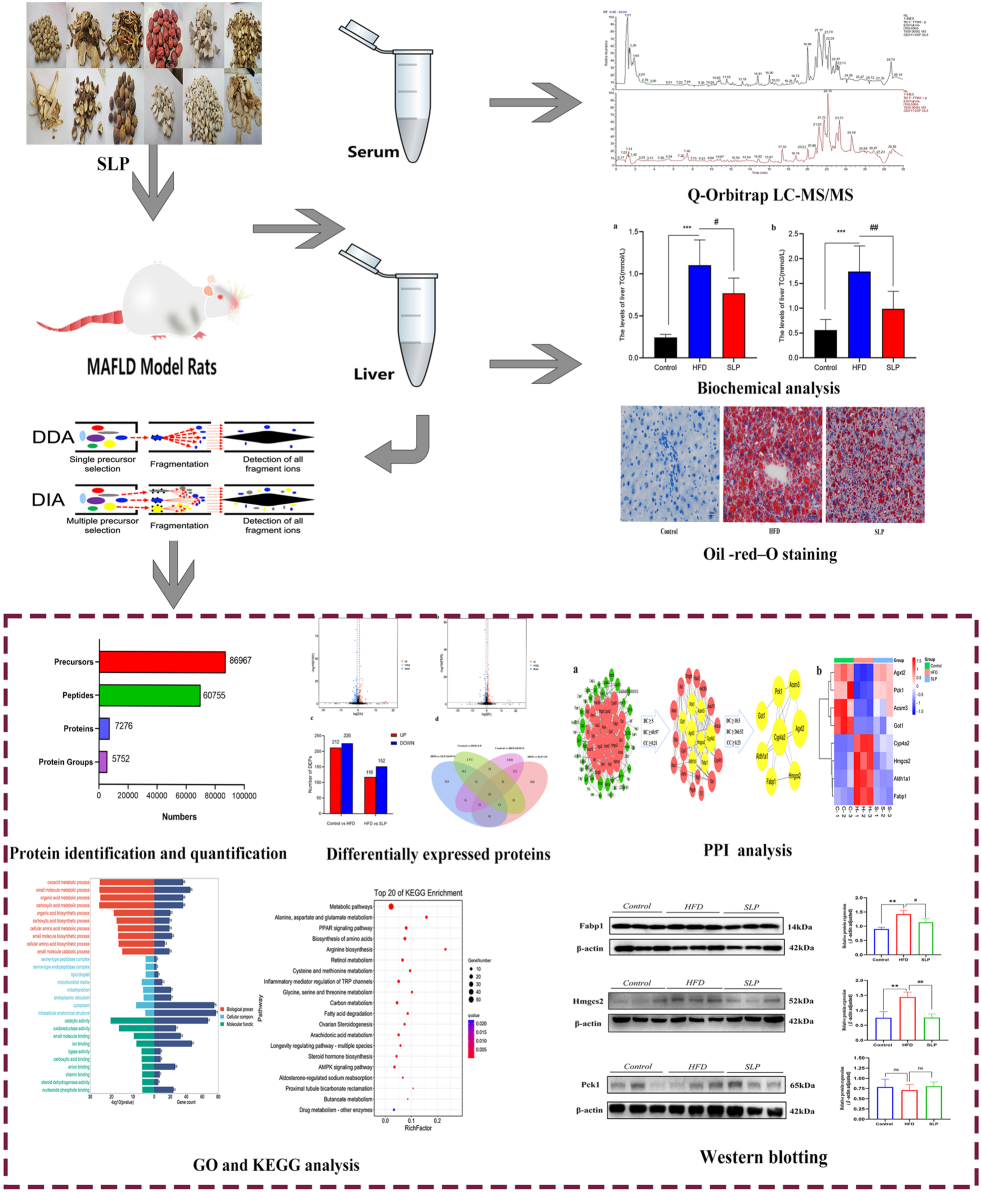
The Experimental flow chart

The whole experimental procedure is shown in the Fig. 1.

### Q-Orbitrap LC–MS/MS determination of the incoming blood components of SLP

Identification and separation of blood components of SLP were performed by Q-Orbitrap LC–MS/MS system (Thermo Fisher Scientific, MA, USA). The chromatographic mass spectrometric setup was consistent with the method according to a previous report [18]. Briefly, the Q-Orbitrap LC–MS/MS was combined with an electrospray ion source in positive and negative ion mode. Chromatographic separations were performed on an UltiMate 3000 RS column (AQ-C18, 150 × 2.1 mm, 1.8 µm, Welch) (Thermo Fisher Scientific, MA, USA) with an oven temperature of 35 °C and a flow rate of 0.3 ml/min; 0.1% formic acid aqueous solution (A)-methanol; (B) as mobile phase, using a gradient elution. The Q-Orbitrap LC–MS/MS acquisition data were initially classified using CD2.1 (Thermo Fisher Scientific, MA, USA) followed by mzCloud database (https://www.mzcloud.org/) search for comparison.

### Biochemical analysis

Liver was mixed with anhydrous ethanoland homogenized at low temperature, centrifuged for 10 min (2500 rpm/min) and then the supernatant was extracted for e determination of total cholesterol (TC) and triglyceride (TG) (Nanjing Jiancheng Institute of Biological Engineering, Nanjing, CN).

### Oil red O staining

Experiments were performed according to the instructions of the oil red O staining kit (BioCloud Biotechnology, Shanghai, CN). Briefly, frozen liver sections were dried, fixed in 10% formaldehyde solution for 10 min, washed with washing solution and then immersed in diluted oil red O staining solution for 20 min, washed in washing solution and stain with hematoxylin (Meyer) for 2 min, followed by tap water rinsed for 5 min, ddH_2_O washed for 10 s and sealed with glycerol gel. Finally, observed and photographed under microscope.

### Data-independent acquisition (DIA) proteomics

#### Protein extraction and digestion

Liver tissue was ground to powder in liquid nitrogen, added to protein lysis solution (7 M urea, 2% SDS, 0.1% PMSF, 65 mM DTT) and lysed by sonication. After centrifuging at 4 °C for 30 min (14,000 rpm/min), the precipitate was discarded, and the supernatant was measured for protein content. 50 μg protein was mixed with 1 μL 1 M DTT and incubated at 55 °C for 1 h; 5 μL 1 M iodoacetamide was added and left for 1 h in a dark place; mixed with 300 μL pre-cooled acetone and precipitated at − 20 °C for 2 h. Finally, the precipitate and Trypsin (Promega, Madison, WI) were mixed in a 50:1 ratio and left to digest overnight at 37 °C.

#### High PH reverse phase separation

All sample peptide mixtures were redissolved in buffer A (buffer A: 20 mM ammonium formate in water, adjusted to pH 10.0 with ammonia) and an Ultimate 3000 system (Thermo Fisher Scientific, MA, USA) was connected to a reverse column (XBridge C18 column, 4.6 mm × 250 mm, 5 μm, (Waters Corporation, MA, USA)) for high pH separation. High pH separation conditions were set to a linear gradient from 5 to 45% B in 40 min (B: 80% ACN with 20 mM ammonium formate, adjusted to pH 10.0 with ammonia). The column was equilibrated for 15 min under initial conditions, with the flow rate maintained at 1 mL/min and the temperature at 30 °C. Six fractions were collected, and each fraction was dried in a vacuum concentrator for use.

#### Data-dependent acquisition (DDA) analysis and DIA analysis

The desalted lyophilized peptide was re-dissolved into a suspension by adding 30 μL buffer A (0.1% formic acid solution). 9 μL suspension was mixed with 1 μL 10 × iRT and analyzed by on-line nanospray LC-MS/MS on an Orbitrap Fusion Lumos coupled to EASY-nLC 1200 system (Thermo Fisher Scientific, MA, USA). The 3 μLpeptide sample was transferred to an analytical column (Acclaim PepMap C18, 75 μm × 25 cm) and separated with a 120 min gradient separation, from 5 to 35% B (B: 0.1% formic acid ACN solution). The column flow rate was maintained at 200 nL/min and the column temperature at 40 °C. The electrospray voltage was maintained at 2.0 kV throughout the process. The peptides were separated by chromatography and then detected by an Orbitrap Lumos mass spectrometer (Thermo Fisher Scientific, MA, USA) in DDA mode. The specific parameters were as follows: (1) MS: scan range = 350–1500(m/z); resolution = 120,000; AGC target = 4e5; Maximum injection time (MIT) = 50 ms; Dynamic exclusion time (DET) = 30 s; (2) HCD-MS/MS: Resolution = 15,000; AGC target = 5e4; MIT = 35 ms; Collision energy (CE) = 32.

The liquid chromatography conditions for DIA were the same as those for DDA. The Orbitrap Lumos mass spectrometer takes a DIA approach, and the DIA MS parameters were set as follows: (1) MS: Scan range = 350–1500 (m/z); Resolution = 120,000; AGC target = 4e6; MIT = 50 ms; (2) HCD-MS/MS: Resolution = 30,000; AGC target = 1e6; CE = 32; stepped CE = 5%. (3) Variable window acquisition with 60 windows set up and overlapping serial ports set up with 1 m/z overlap per window.

#### Database search and analysis

The raw DDA data was combined and analyzed by Spectronaut Pulsar X and a database of spectra was created. Trypsin was set up for enzymatic digestion, while allowing for two amino acid miss-sites. Library search parameters: the fixed modification: carbamidomethylation; the variable modification: methionine oxidation. Library standards are 1% precursor false discovery rate(FDR), 1% protein FDR, 1% peptide FDR (Additional file 1: File S1).

The raw data from DIA were processed and analyzed by Spectronaut X (Biognosys AG, Switzerland). The qualitative criteria for protein were a precursor threshold of 1.0% FDR and a protein threshold of 1.0% FDR. The peak areas of the first three MS1 peptides with FDR ≤ 0.01 were chosen as the criteria for protein quantification.

#### Bioinformatics analysis

Proteins were annotated against the gene ontology (GO, http://www.geneontology.org) and kyoto encyclopedia of genes and genomes (KEGG, https://www.genome.jp/kegg/) databases to obtain their function. Any absolute fold change > 1.5 and Q value < 0.05 was considered differentially expressed proteins (DEPs). DEPs with overlapping but opposite expression trends between the two comparison groups (control group vs. HFD group and HFD group vs. SLP group) were screened and then enrichment analyses were performed on those by the OmicShare tool (https://www.omicshare.com/tools).When*p* < 0.05 for a KEGG pathway or a GO entry, which indicates these DEPs are significantly enriched in the KEGG pathway or GO entry.

#### Building protein–protein interaction (PPI) networks

These DEPs, with overlap and opposite expression trends, were uploaded to the STRING database (http://www.string-db.org/) for PPI network analysis. The nodes of PPI network need to interact with each other in order to function biochemically, and the interaction score represents the degree of association between nodes and nodes. Having too low an interaction score between nodes means that there are more interactions and more false positives. To avoid more false positives, the minimum required interaction score was set to a medium confidence level (0.400) and the unconnected nodes in the network were hidden. The TSV files was downloaded and uploaded to Cytoscape 3.7.2. CytoNCA is a plugin for Cytoscape, evaluation and visualization analysis for multiple centrality measures [19]. CytoNCA was used to obtain the topological parameters of each node in the network: including “degree centrality (DC)”, “betweenness centrality (BC)”, “closeness centrality (CC)”. The definitions and formulae for the calculation of these parameters have been defined previously [20]. Briefly, the higher the scores of “DC”, “BC” and “CC”, the more central and important the node is in the network. In order to obtain core DEPs, nodes with scores above the median of DC, BC and CC were generally considered to be important.

### Western blotting analysis

Added 0.4 ml RIPA buffer (P0013B, Beyotime Biotechnology, CN) containing protease inhibitor (P1050, Beyotime Biotechnology, CN) to 40 mg rat liver tissue, homogenized the liver by tissue grinder (KZ-III-FP, Servicebio, CN), placed on ice for 1 h, centrifuged at 4 °C for 10 min (12000 rpm/min), and collected the supernatant. Protein concentration was determined using the BCA kit (P10012S, Beyotime Biotechnology, CN), samples were mixed with SDS-PAGE protein loading buffer (5 ×) (P0015, Beyotime Biotechnology, CN) in a 4:1 ratio and placed in dry bath incubator at 100 °C for 5 min to denature the proteins. The proteins were separated on 8% or 12% SDS-PAGE gel, containing 20–45 ug of protein per lane. Afterwards, the separated proteins were transferred to PVDF membranes (FFP24, FFP32, Beyotime Biotechnology, CN), which were then blocked in 5% skimmed milk for 1.5 h. After blocking, the PVDF membranes were washed three times with TBST, then rabbit anti-Fabp1 (1:2000, 32,764–1, Signalway Antibody, USA), rabbit anti-Hmgcs2 (1:2000, 29,188–1, Signalway Antibody, USA), rabbit anti-Pck1 (1:2000, 41,644–1, Signalway Antibody, USA) and rabbit anti-β-actin (1:2000, GB11001, Servicebio, CN) were added and incubated overnight at 4 °C. After incubation, PVDF membranes were washed three times with TBST and incubated with HRP-conjugated secondary antibodies (1:10,000, G1213, Servicebio, CN) for 1 h at room temperature. The membranes were washed against with TBST and then immunoreactivity was determined using enhanced BeyoECL chemiluminescence reagent (P0018AS, Beyotime Biotechnology, CN) and analyzed with Image J.

### Statistical analysis

All data were obtained from at least three independent experiments and data were statistically analyzed using GraphPad Prism 8.0, with results expressed as mean ± standard deviation ($\overline{x }$ ± SD). Data between groups were analyzed using Student's t-test and one-way ANOVA. *p* < 0.05 was considered statistically significant.

## Results

### Chromatographic fingerprint analysis

**Fig. 2:**
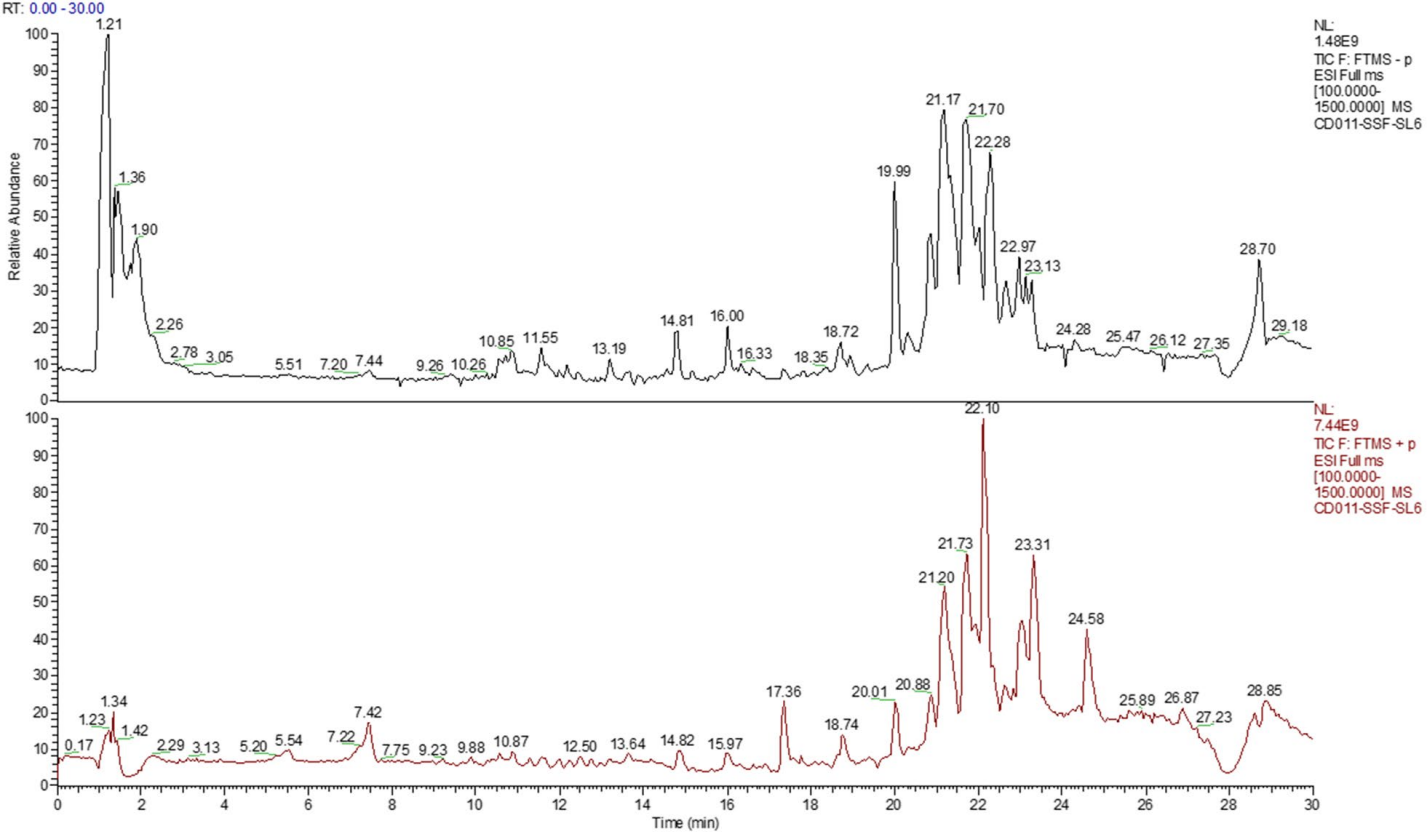
Q-Orbitrap LC–MS/MS TICs of SLP-containing serum

Q-Orbitrap LC–MS/MS was used to identify the incoming blood components of SLP. The Fig. 2 shows the total ion chromatograms (TICs) of the components in SLP-containing serum. In mzCloud, SLP was matched to 709 compounds, the control serum to 314 compounds and the SLP-containing serum to 299 compounds. A total of 18 incoming blood components of SLP were identified, of which 10 compounds scored > 60 on the mzCloud best match (Table 1).

**Table 1:** The incoming blood components of SLP-containing serum

	Name	Formula	RT [min]	mzCloud best match
1	18-β-Glycyrrhetinic acid	C30 H46 O4	21.104	95.8
2	Methyl palmitate	C17 H34 O2	15.432	93.2
3	( ±)-Abscisic acid	C15 H20 O4	15.369	87.3
4	2-Hydroxymyristic acid	C14 H28 O3	18.359	85
5	(3β,5ξ,9ξ)-3,23-Dihydroxy-1-oxoolean-12-en-28-oic acid	C30 H46 O5	19.233	75.6
6	Methyl 3,5-di-tert-butyl-4-hydroxybenzoate	C16 H24 O3	17.561	72.9
7	17α-Methyl-androstan-3-hydroxyimine-17β-ol	C20 H33 N O2	19.109	70.9
8	2-[(1S,4S,5S)-4-{[(Cyclohexylcarbamoyl)amino]methyl}-5-isopropyl-2-methyl-2-cyclohexen-1-yl]-N-[2-(dimethylamino)ethyl] acetamide	C24 H44 N4 O2	20.582	65.9
9	CP 47,497-C8-Homolog C-8-hydroxy metabolite	C22 H36 O3	20.881	64.6
10	Andrographolide	C20 H30 O5	17.148	62.9
11	Δ17-6-keto prostaglandin F1α	C20 H32 O6	17.01	44
12	( +)-Syringaresinol	C22 H26 O8	10.684	41.7
13	1,1’-(2,6-Dimethyl-4-(3-nitrophenyl)-1,4-dihydropyridine-3,5-diyl) diethanone	C17 H18 N2 O4	16.108	37.2
14	Isorhapontigenin	C15 H14 O4	14.862	31.2
15	N3-(2-Mercapto-4-oxo-3,4-dihydroquinazolin-3-yl)nicotinamide	C14 H10 N4 O2 S	29.772	42.8
16	N-Acetyldopamine	C10 H13 N O3	8.068	32.8
17	Piceatannol	C14 H12 O4	15.608	42.9
18	Simazine	C7 H12 Cl N5	7.232	42.6

### Biochemical analysis

**Fig. 3:**
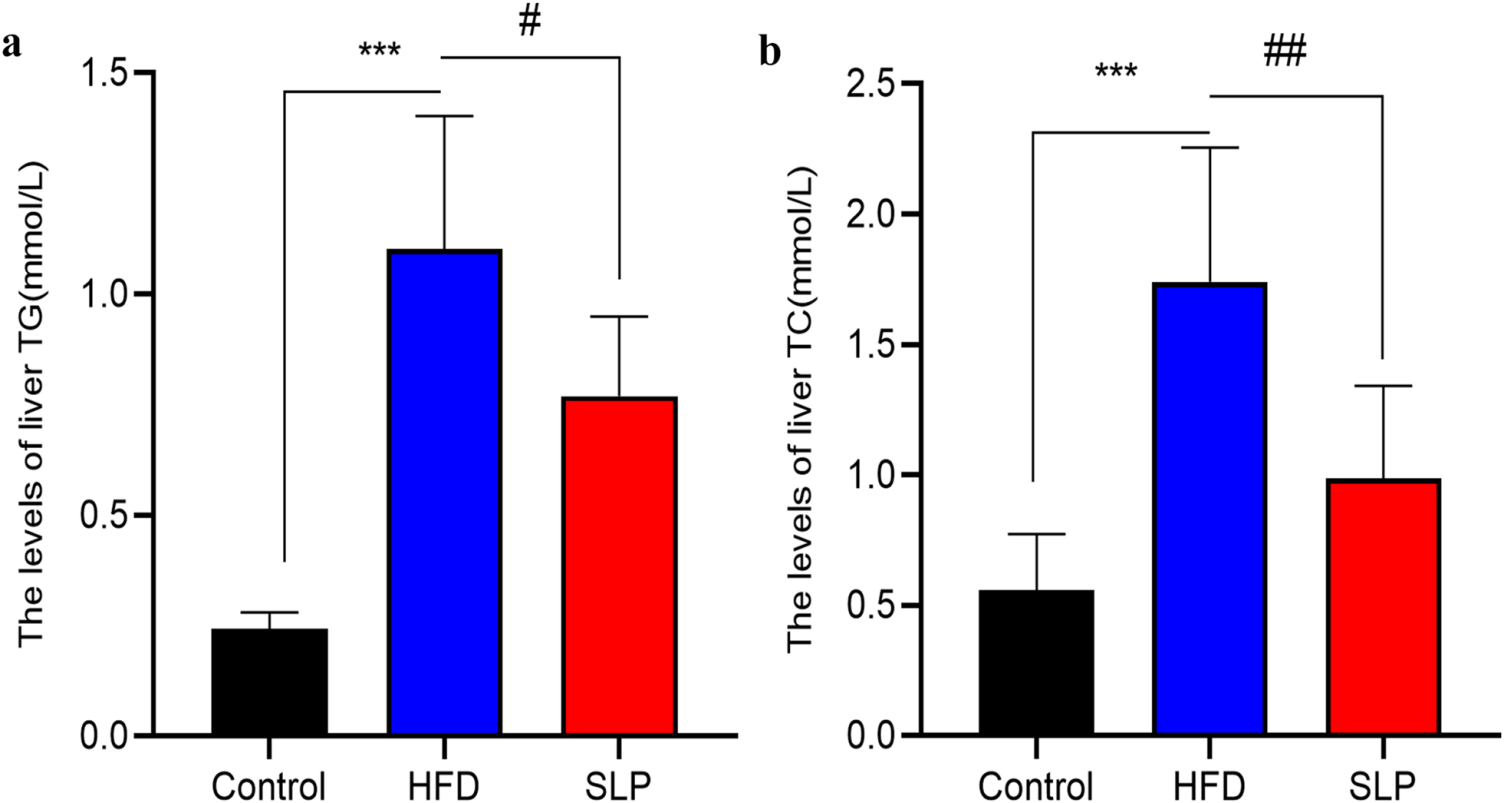
Comparison of liver lipids ($\overline{x }$± SD, n = 6). Note: control group vs. HFD group, ^***^p < 0.001. HFD group vs. SLP group, ^#^*p* < 0.05, ^##^*p* < 0.01.)

SLP was able to regulate lipid levels in MAFLD rats. The Fig. 3 shows that TC and TG levels were increased in MAFLD rats compared to healthy rats. After 3 weeks of treatment with SLP, the levels of TC and TG in MAFLD rats were significantly reduced.

### Oil red O staining

**Fig. 4:**
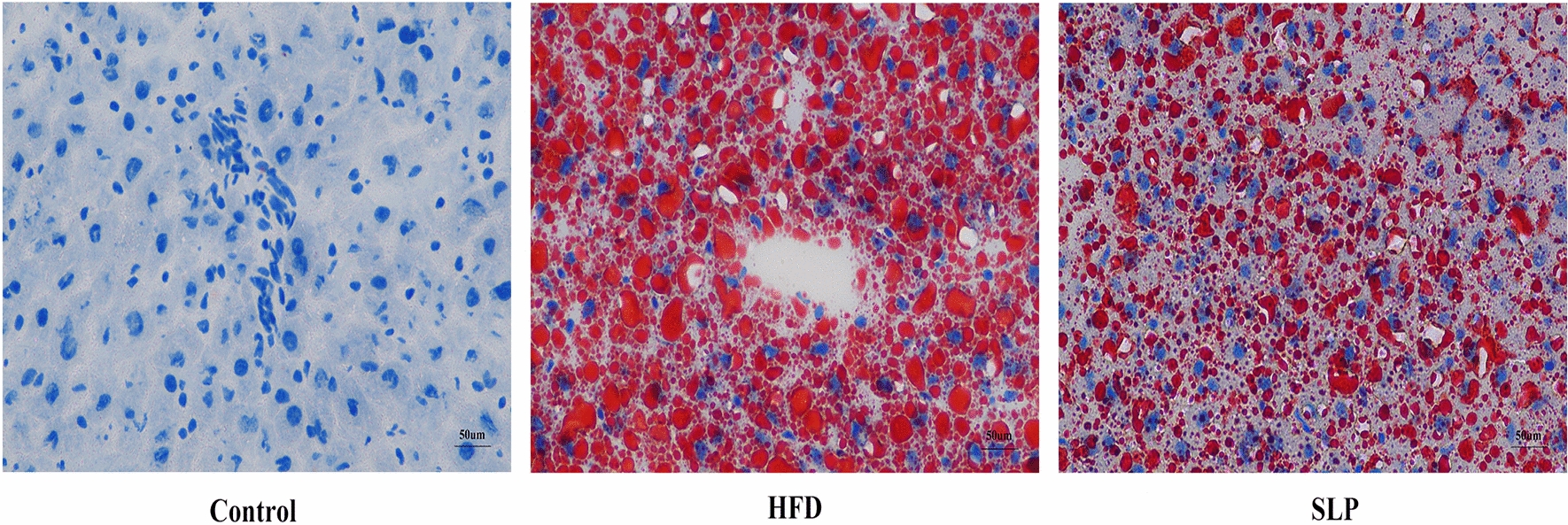
The oil red O staining results. (Red is the lipid droplet, blue is the nucleus, the picture is magnified 400 times)

Based on the observation of the oil red O staining in Fig. 4, we can find that SLP has the effect on improving lipid accumulation in liver. We did not observe red lipid droplets formation in control group, in contrast, many red lipid droplets were produced and fused into sheets in HFD group. Compared to HFD group, the red lipid droplets in the rat liver of SLP group was significantly reduced in number, smaller in shape and lighter in color after 3 weeks of SLP treatment.

### Proteome analysis

**Fig. 5:**
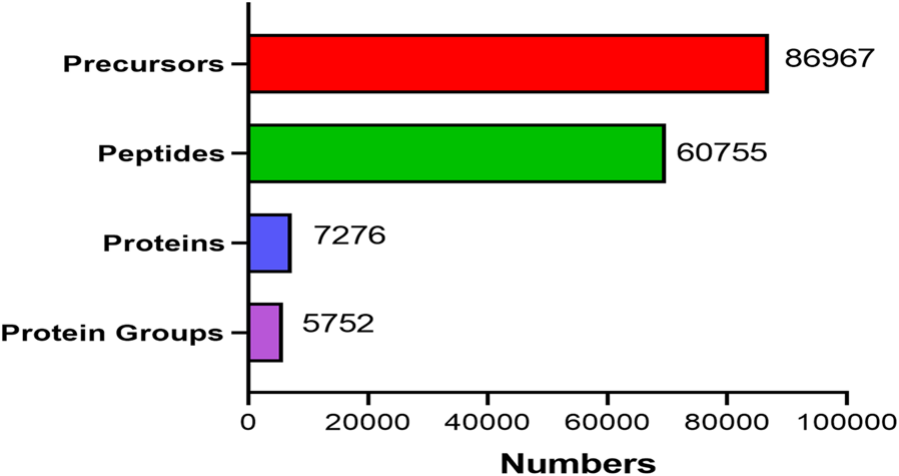
Statistical picture of identification and quantitative results

**Fig. 6:**
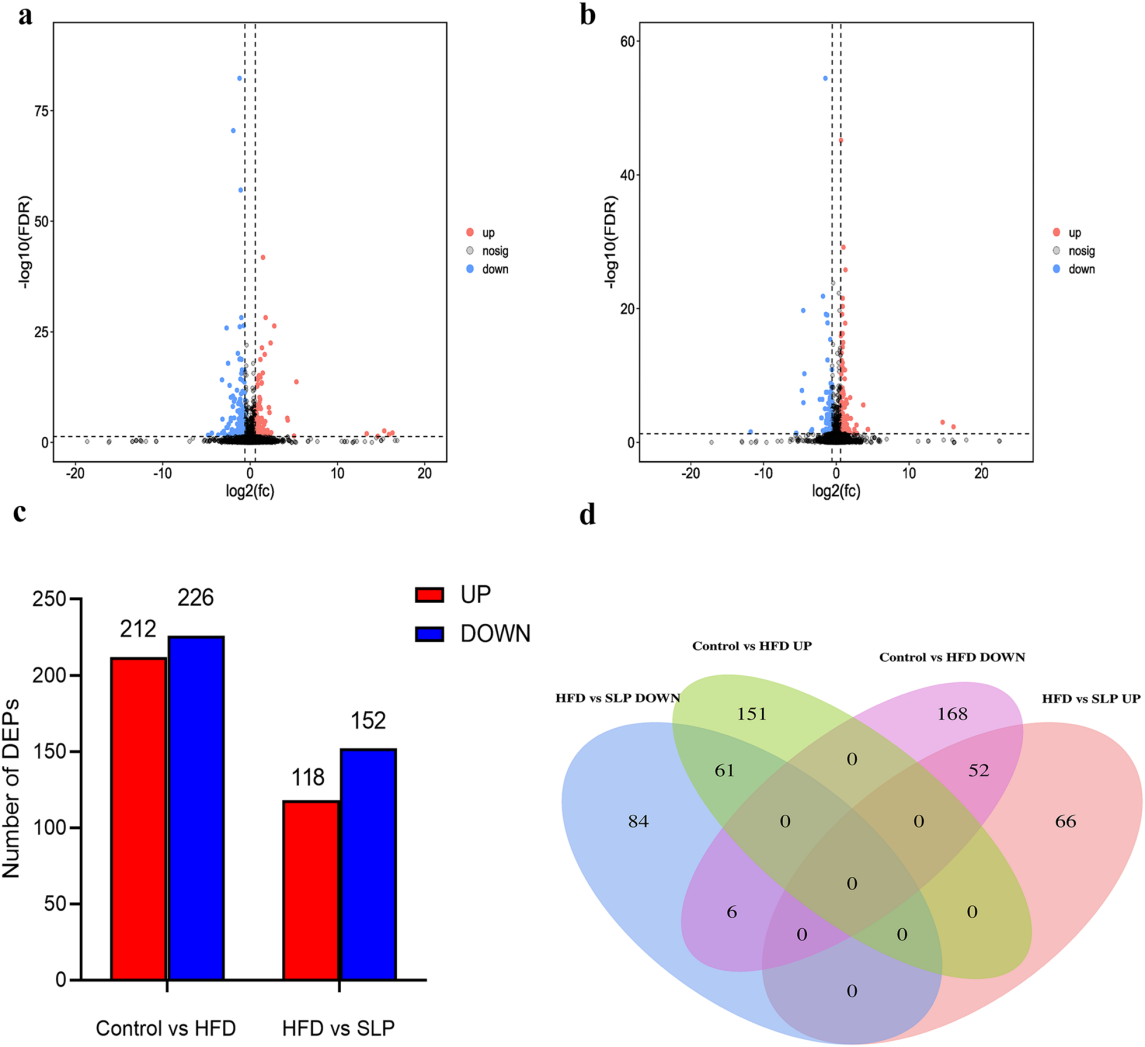
DEPs among the three groups. Volcano plot, **a** DEPs in control group vs. HFD group, volcano plot; **b** DEPs in HFD group vs. SLP group. up-regulated proteins are shown in red, down-regulated proteins are shown in blue, and black dots indicate no difference. **c** The bar chart shows the number of DEPs in the two comparison groups. **d** Venn diagram showing overlapping DEPs of the two comparison groups

A total of 86,967 precursors were obtained by DIA quantitative proteomics analysis of all samples, and a total of 60,755 peptides were identified and 7276 proteins were quantified according to a filtering criterion of FDR ≤ 0.01, of which 5752 protein matches were highly similar (Additional file 2: File S2, Additional file 3: File S3, Fig. 5). Proteins with significant differences between groups were screened according to the absolute value of fold change > 1.5 and Q value < 0. 05. The volcano plot (Fig. 6a, b) show that there were 438 DEPs in control group vs. HFD group, of which 212 had increased expression and 226 had decreased expression (Additional file 4: File S4, Fig. 6a); compared to HFD group, SLP group had 118 DEPs with increased expression and 152 DEPs with decreased expression (Additional file 5: File S5, Fig. 6b). A total of 119 DEPs overlapped between the two comparison groups (control group vs. HFD group and HFD group vs. SLP group),, of which 113 DEPs were expressed in an opposite trend (Additional file 6: File S6, Fig. 6d), indicating that these 113 DEPs are potential for SLP against MAFLD and are proteins that need further analysis in our study.

### GO and KEGG pathway enrichment analyses

**Fig. 7:**
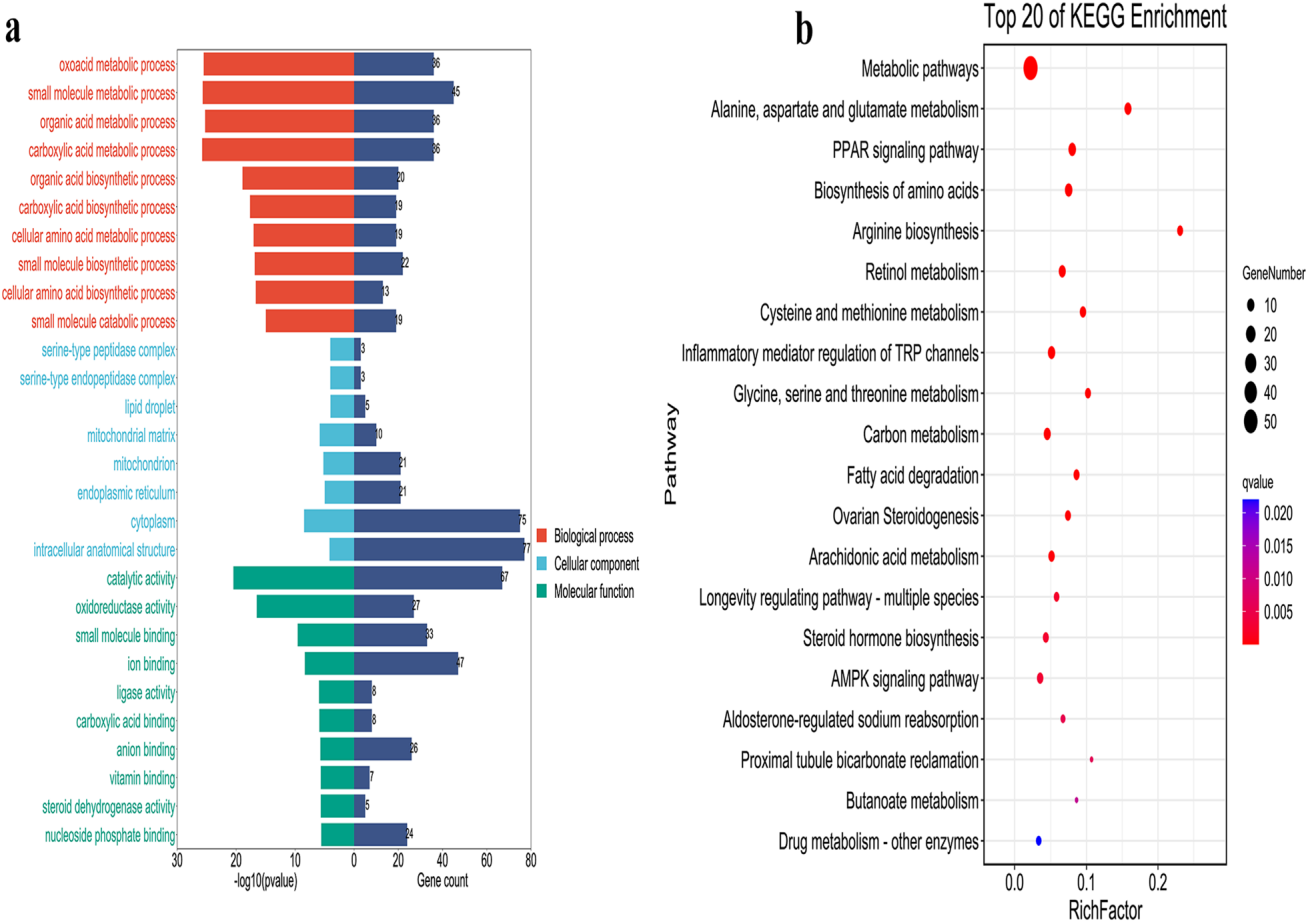
Bioinformatics analysis of DEPs with overlapping and opposing expression trends in the two comparison groups. **a** GO functional enrichment analysis including biological processes, cellular components and molecular functions. **b** KEGG enrichment pathway analysis

The GO functional enrichment analysis of these 113 DEPs is shown in Fig. 7a. A total of 25 biological processes were enriched (*p* < 0.05), including oxoacid metabolic, organic acid metabolic, carboxylic acid metabolic, small molecule metabolic and other biological processes; they were mainly expressed in cellular components such as cytoplasm, mitochondrial matrix and endoplasmic reticulum, and were able to perform molecular functions such as catalytic activity and oxidoreductase activity (Fig. 7a). It is worth noting that the DEPs in both comparison groups are involved in oxoacid metabolic, lipid metabolic, carboxylic acid metabolic and small molecule metabolic processes, and both have molecular functions such as catalytic activity and hydrolase activity. DEPs in control group vs. HFD group were enriched in cellular fractions such as the lipid particle, endoplasmic reticulum membrane and organelle membrane, whereas DEPs in HFD group vs. SLP group were mainly expressed in cellular fractions such as the lipid particle, peroxisome, autolysosome, secondary lysosome, and mitochondrion (Additional file 7: File S7, Additional file 8: File S8, Additional file 11: Fig. S1, Additional file 12: Fig. S2). It is suggested that SLP is involved in processes such as oxidative metabolism and lipid metabolism by regulating the activity of related proteins on mitochondria and endoplasmic reticulum to improve lipid deposition and inflammatory infiltration in MAFLD.

KEGG annotation and pathway analysis will help us to better understand how DEPs orchestrate their biological functions, so we used KEGG to annotate the identified proteins at the biological pathway level, which allowed us to identify the biochemical metabolic pathways and signal transduction pathways in which these proteins are primarily involved. The 113 DEPs were mainly enriched to 52 signaling pathways (*p* < 0.05) and most of these pathways were closely associated with impaired energy metabolism. As shown in Fig. 7b, these include amino acid metabolic pathways (glycine, serine and threonine metabolism, alanine, aspartate and glutamate metabolism, etc.), lipid metabolic pathways (PPAR signaling pathway, fatty acid degradation, etc.) and oxidative stress pathways (arachidonic acid metabolism, retinol metabolism, etc.). Meanwhile, we found that the pathways that DEPs were enriched to in both comparison groups were largely the same, for example PPAR signaling pathway, arachidonic acid metabolism and glycine, serine and threonine metabolism were significantly enriched, suggesting that they may be key pathways for SLP treatment of MAFLD (Additional file 9: File S9, Additional file 10: File S10, Additional file 13: Fig. S3, Additional file 14: Fig. S4).

### PPI analysis

**Fig. 8:**
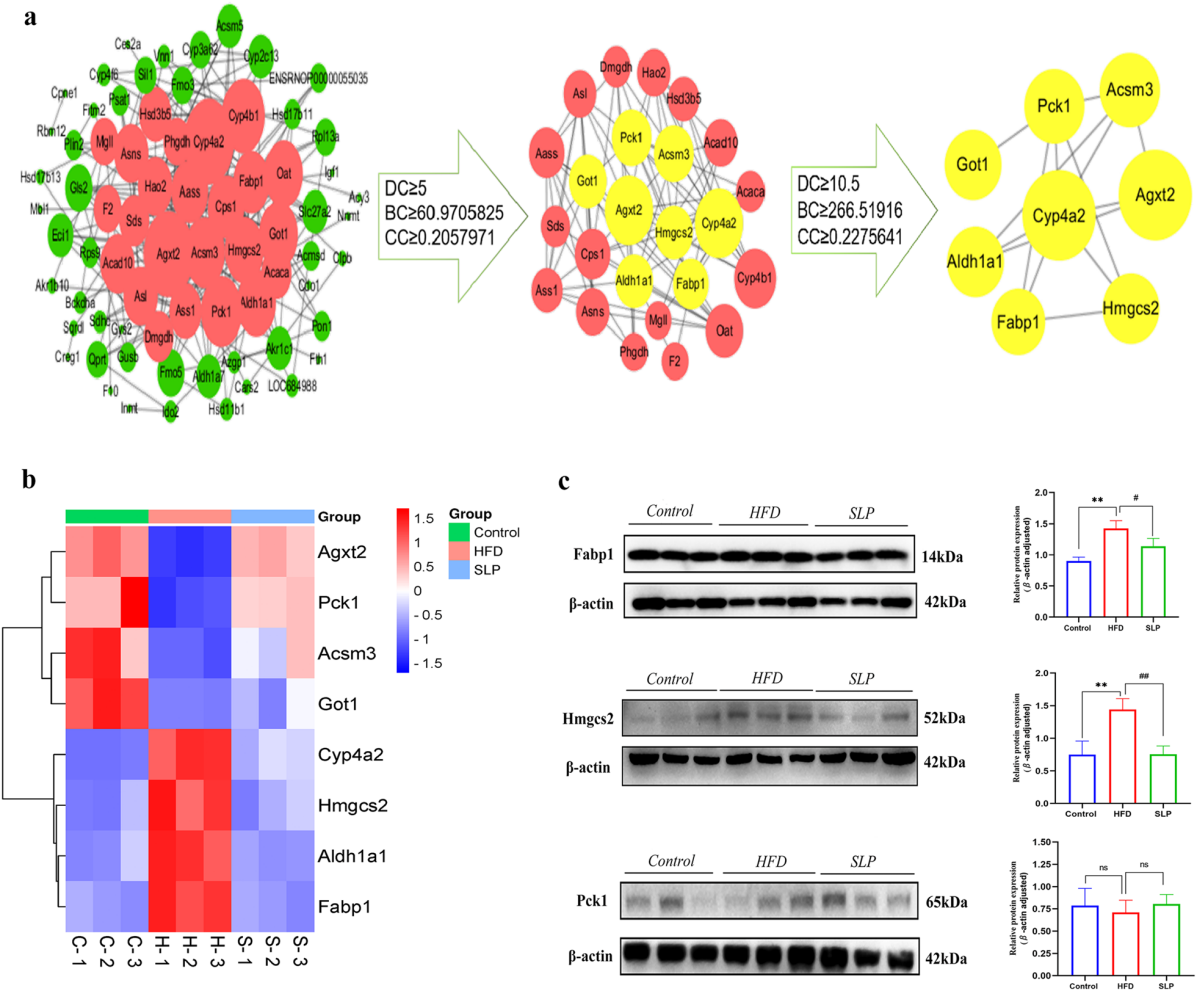
**a** PPI analysis and topology analysis of 113 DEPs, and 2 sub-networks created based on topology analysis results. **b** Hierarchical clustering diagram of the core differential proteins. **c** Western blotting analysis validation of the expression of 3 proteins, Hmgcs2, Fabp1 and Pck1. control group vs. HFD group, *p < 0.05. **p < 0.01, HFD group vs. SLP groups, ^#^*p* < 0.05, ^##^*p* < 0.01

The Fig. 8a is show that the PPI network with 72 nodes and 207 edges. The PPI results were topologically analyzed with the first filter set to DC ≥ 5, BC ≥ 60.9705825 and CC ≥ 0.2057971, yielding a total of 24 nodes and 86 edges. These 24 nodes may be key DEPs for SLP treatment of MAFLD, and we performed further screening on them. The second screening condition was set as: DC ≥ 10.5, BC ≥ 266.51916, CC ≥ 0.2275641, there were 8 key nodes and 14 edges obtained, namely alanine-glyoxylate transaminase 2 (Agxt2), cytochrome P450 4a2 (Cyp4a2), fatty acid binding protein 1 (Fabp1), 3-hydroxy-3-methylglutaryl-CoA synthase 2 (Hmgcs2), aldehyde dehydrogenase 1A1 (Aldh1a1), phosphoenolpyruvate carboxylase 1 (Pck1), acyl-CoA synthase medium chain 3 (Acsm3) and Glutamic-oxaloacetic transaminase 1 (Got1). The expression of these eight proteins in the different groups is shown in Fig. 8b. Fabp1, Aldh1a1 and Acsm3 are key enzymes in lipid anabolism, Pck1 and Got1 are involved in gluconeogenesis, Hmgcs2 is a ketogenic rate limiting enzyme and Cyp4a2 is a fatty acid ω-hydroxylase, all involved in fatty acid oxidation. Agxt2 is an amino acid transferase that promotes the formation of antioxidant enzymes. The results showed that SLP was able to inhibit lipid synthesis and lipid peroxidation and restore the oxidative balance of the liver.

### Western blotting analysis

To validate the proteomics results, we randomly selected three animal livers from each group and randomly selected 3 core DEPs were verified by western blotting analysis. As shown in Fig. 8c, the expression of Hmgcs2 and Fabp1 proteins were significantly increased (*p* < 0.01) and the expression of Pck1 protein was decreased (*p* > 0.05) in HFD-induced MAFLD rats compared to the control group. After 3 weeks of treatment, the expression of Hmgcs2 and Fabp1 proteins significantly decreased (*p* < 0.05) and the expression of Pck1 protein increased (*p* > 0.05). The results of the DIA analysis were confirmed.

## Discussion

Abnormal lipid accumulation in hepatocytes is the fundamental pathological feature of MAFLD, and inflammation due to disturbances in hepatic lipid metabolism is important cause of MAFLD progression. When hepatocytes continue to accumulate large amounts of lipids, lipid peroxidation leads to the production of large amounts of reactive oxygen species (ROS) and highly toxic free fatty acids, causing a series of inflammatory changes such as oxidative stress and inflammatory factor accumulation, leading to hepatocellular damage [21]. TCM believes that lipids are one of the manifestations of nutrients and that the transport of nutrients in the body depends on the proper functioning of the “spleen qi dispersing essence” function, which, when impaired, leads to abnormal retention of fat in the liver and triggers MAFLD. As a representative formula of “spleen qi dispersing essence”, SLP is widely used in the treatment of metabolic diseases. In this study, the active therapeutic components in SLP were identified using Q-Orbitrap LC–MS/MS (Table1). Available studies suggest that several of these chemical components are beneficial in reducing blood lipids. 18β-Glycyrrhetinic acid maintains very low density lipoprotein (VLDL) homeostasis and inhibits gluconeogenesis by suppressing hepatocyte nuclear factor 4α expression [22] and protects hepatocytes by competitively inhibiting cytochrome P450 (Cyp450) [23]. Isorhapontigenin reduces lipids by regulating the activity and stability of PPARγ in adipocytes [24]. Andrographolide reduces blood lipid levels and enhances insulin sensitivity by inhibiting fatty acid production and promoting fatty acid oxidation [25, 26]. It shows that SLP does regulate fatty acid metabolism, and from an objective point of view, it clarifies the connotation of the theory that SLP plays a role in “dispersing the essence of spleen”.

DIA technology has the advantages of panoramic scanning, ultra-high reproducibility, stability, and accuracy, as well as the ability to analyze data retrospectively, making it suitable for large sample size analysis. We used DIA proteomics to identify 438 DEPs in control group vs. HFD group, 270 DEPs in HFD group vs. SLP group. Between the two comparison groups (control group vs. HFD group and HFD group vs. SLP group). 113 DEPs were found to be expressed in opposite trends and are therefore considered to be key proteins for SLP in the treatment of MAFLD. Both GO and KEGG enrichment results show that these DEPs are closely associated with lipid metabolism and oxidative stress. In addition, eight core DEPs identified by PPI topology analysis will be discussed in detail, including Fabp1, Acsm3, Aldh1a1, Pck1, Got1, Hmgcs2, Cyp4a2 and Agxt2.

Aldh1a1 catalyzes the oxidation of retinal to retinoic acid (RA). Expression of Aldh1a1 was significantly increased in the livers of patients and rats with NAFLD [27, 28]. Inhibition of RA inhibited weight gain, gluconeogenesis and TG accumulation in NAFLD rats [29, 30], but it is noteworthy that RA levels in MAFLD patients were negatively correlated with the degree of fat accumulation [31], which is inconsistent with the results of animal studies, considering the possibility of differences in gene expression between species. Ma et al. [32] suggested that the effect of Aldh1a1-induced RA content on NAFLD depends on whether it activates retinoic acid receptors (RARs) or retinoic acid X receptors (RXRs), and that RARs and RXRs have opposite effects on lipid regulation. This may be why inhibition of retinoic acid levels can alleviate altered metabolic phenotypes. Acsm3 is an acyl coenzyme A synthase that interacts with medium-chain fatty acids on the outer mitochondrial membrane to participate in fatty acid β-metabolism, and reduced Acsm3 leads to fatty acid accumulation [33]. Previous studies have shown that Fabp1 is a key regulator of hepatic lipid metabolism [34], involved in the uptake of long-chain fatty acids and promoting the production and secretion of hepatic triacylglycerol and VLDL [35]. Overexpression of Fabp1 exacerbates hepatic steatosis [36], while hepatic lipid accumulation and oxidative stress are significantly improved after knockdown of Fabp1 [32, 37], our data also supports this result. In this study Fabp1 and Aldh1a1 expression was increased and Acsm3 expression was decreased in the model group, and SLP intervention was able to reverse this alteration. Combined with the results of GO and KEGG, we have reason to believe that SLP can inhibit lipid synthesis.

In response to the abnormal elevation of fatty acids, the body's fatty acid oxidation reactions are enhanced. The Cyp450 enzyme system is a family of mixed function oxidative enzymes, mostly expressed in the liver, and about 75% of all drugs in the body are metabolized by Cyp450 [38]. Cyp4a2 is positively correlated with lipid peroxidation levels [39]. Cyp4a2 is an ω-oxidase that metabolizes fatty acids via ω-oxidation to produce dicarboxylated fatty acids, ROS, which impairs mitochondrial function and leads to oxidative stress and hepatocyte damage [40], and lowering Cyp4a2 can restore damaged hepatocytes [39, 41]. TG formation is closely related to the amount of non-esterified fatty acids (NEFAs). In the liver, NEFAs are broken down to acetyl coenzyme a by mitochondrial β-oxidation, which then forms ketone bodies via the ketogenic pathway or enters the TCA cycle for oxidation to form CO_2_ and is excreted from the body [42]. The Hmgcs2 enzyme is the only key regulator of the ketogenic pathway, and the body compensates by enhancing ketogenesis, allowing excess fatty acid metabolism to be broken down into ketone bodies, thereby reducing the load on the liver [43–45]. Although ketogenesis has been shown to be effective in reducing lipid formation, in a prolonged ‘ketosis’year environment, the body restricts ketogenesis to prevent the accumulation of large amounts of ketone bodies leading to acidosis [42]. Recent studies [46] have shown that Hmgcs2 is upregulated in expression in NAFLD mouse models, while Hmgcs2 deficiency and inhibitors promote ketogenesis in NAFLD mice and palmitate induced HepG2, attenuate hepatocyte injury and inflammation due to lipotoxicity. Thus, acetyl coenzyme a is more likely to enter the TCA cycle for oxidation [47]. The hepatic TG content of NAFLD patients is positively correlated with TCA cycling flux, and excessive mitochondrial TCA cycling leads to abnormally high oxidative fluxes and ROS levels [48], leading to mitochondrial damage, triggering oxidative stress and exacerbating hepatic mitochondrial dysfunction. Agxt2 is a mitochondrial aminotransferase that catalyzes the conversion of glyoxylic acid to glycine [49]. Glycine is the raw material for the synthesis of glutathione, which has the function of scavenging oxygen free radicals and reducing the degree of lipid peroxidation [50]. Low expression of Agxt2 leads to insufficient glycine biosynthesis [51], imbalance of antioxidant and oxidative status of the liver, resulting in hepatic lipid deposition-induced endoplasmic reticulum stress, and exacerbating fibrotic changes in NAFLD mice [52]. In the present study, Cyp4a2 and Hmgcs2 were highly expressed in the HFD group, mediating excessive mitochondrial oxidation and a large accumulation of ROS. The inhibition of Aext2 expression in the HFD group contributed to the lack of antioxidant enzyme levels, leading to a breakdown of the dynamic balance between oxidation and antioxidation and further inducing oxidative stress and mitochondrial disorders. After SLP intervention, it was able to inhibit the increase of Cyp4a2 and Hmgcs2 and the decrease of Aext2 expression, indicating that SLP can improve oxidative stress in MAFLD rats by regulating oxidative balance. Abnormally enhanced glycolysis accelerates the catabolism of glucose to pyruvate, which can be oxidatively decarboxylated to acetyl coenzyme A to enter the TCA cycle, exacerbating mitochondrial damage of MAFLD [53], and inhibition of glycolysis can effectively prevent lipotoxic damage to liver cells [54, 55]. Got1 is an amino acid metabolism enzyme, which has been shown to interact with pyruvate carboxylase (PC) to reduce the oxidative decarboxylation of pyruvate into the TCA cycle in order to reverse the worsening of MAFLD caused by abnormalities in glycolysis [56]. Pck1 is a gluconeogenesis rate-limiting enzyme, and our results found that Pck1 expression was reduced in the liver of rats with HFD-induced MAFLD, which is inconsistent with previous literature reporting that the development of MAFLD is accompanied by increased gluconeogenesis [57, 58]. It has been found that gluconeogenesis is the reverse process of glycolysis, and overexpression of Pck1 can activate gluconeogenesis to inhibit glycolysis [59]. Therefore, we suspect that low Pck1 expression in the liver of MAFLD rats may be associated with enhanced glycolysis, but this requires further experimental confirmation.

However, there are still limitations to this study. Firstly, our study only tested a single concentration of SLP (10.89 g/kg/d) and did not set up multiple drug concentrations for parallel comparisons, thus making it impossible to assess whether the effects are dose dependent. Secondly, our study focused on exploring the therapeutic effects and mechanisms of SLP on the disease but lacked a control study of normal diet rats receiving SLP, thus ignoring the possible positive or negative effects of SLP on the normal liver. Although the comparative results between the control and SLP treated groups showed that SLP was almost able to restore DEPs to a normal state in the liver of rats ingesting HFD after 3 weeks of intervention treatment.”

## Conclusion

Quantitative proteomic analysis of DIA confirmed the beneficial effects of SLP on MAFLD, and that SLP is involved in the regulation of different target proteins activating multiple pathways together to exert therapeutic effects. SLP inhibited lipid synthesis by down-regulating the expression of Fabp1 and Aldh1a1 and up-regulating the expression of Acsm3. The levels of Cyp4a2 and Hmgcs2 were down-regulated and Agxt2 was up-regulated in the liver after SLP treatment, indicating that SLP improved ketogenic inhibition and increased the antioxidant capacity of the liver to alleviate HFD-induced oxidative stress. Furthermore, SLP upregulated the expression of Got1 and Pck1, suggesting that SLP enhances hepatic gluconeogenesis and attenuates hepatic glycolysis to restore glucose metabolism homeostasis.

### Supplementary Information

**Additional file 1: File S1.** Refer to Database creation results-dia.

**Additional file 2: File S2.** Identification- all_Peptides.

**Additional file 3: File S3.** Quantification-all_Protein.annot.

**Additional file 4: File S4.** CONTROL-vs-MAFLD.filter. annot.

**Additional file 5: File S5.** MAFLD-vs-SLBZ.filter. annot.

**Additional file 6: File S6.** The common DEPs for the two comparison groups.

**Additional file 7: File S7.** CONTROL-vs-MAFLD.GO. level2.

**Additional file 8: File S8.** MAFLD-vs-SLBZ.GO. level2.

**Additional file 9: File S9.** CONTROL-vs-MAFLD.path.

**Additional file 10: File S10.** MAFLD-vs-SLBZ.path.

**Additional file 11: Figure S1.** CONTROL-vs-MAFLD.GO.level2.bar.

**Additional file 12: Figure S2.** MAFLD-vs-SLBZ.GO.level2.bar.

**Additional file 13: Figure S3.** CONTROL-vs-MAFLD.gradient.

**Additional file 14: Figure S4.** MAFLD-vs-SLBZ.gradient.

## Data Availability

The dataset(s) supporting the conclusions of this article is available in the ProteomeXchange Consortium repository, (PXD042049, https://proteomecentral.proteomexchange.org/cgi/GetDataset?ID=PXD042049). All data generated or analysed during this study are included in this published article (and its supplementary information files).

## Abbreviations

SLP: Shenling Baizhu powder
NAFLD: Nonalcoholic associated fatty liver disease
MAFLD: Metabolic associated fatty liver disease
TCM: Traditional Chinese medicine
DEPs: Differentially expressed proteins
DIA: Data-independent acquisition
DDA: Data-dependent acquisition
HFD: High fat diet
Q-Orbitrap LC–MS/MS: Q Exactive Orbitrap high-resolution liquid chromatography coupled with mass spectrometry/mass spectrometry
TC: Total cholesterol
TG: Triglyceride
GO: Gene Ontology
KEGG: Kyoto Encyclopedia of Genes and Genomes
PPI: Protein–protein interaction
DC: Degree centrality
BC: Betweenness centrality
CC: Closeness centrality
VLDL: Very low-density lipoprotein
Agxt2: Alanine-glyoxylate transaminase 2
Cyp4a2: Cytochrome P450 4a2
Cyp450: Competitively inhibiting cytochrome P450
Fabp1: Fatty acid binding protein 1; Hmgcs2: 3-hydroxy-3-methylglutaryl-CoA synthase 2
Aldh1a1: Aldehyde dehydrogenase 1A1
Pck1: Phosphoenolpyruvate carboxylase 1
Acsm3: Acyl-CoA synthase medium chain 3
Got1: Glutamic-oxaloacetic transaminase 1
ROS: Reactive oxygen species
PC: Pyruvate carboxylase
TICs: Total ion chromatograms
NEFAs: Non-esterified fatty acids
RA: Retinoic acid
RXRs: Retinoic acid receptors
RXRs: Retinoic acid X receptors
