# Supplementary figures and images for: Quantitative proteomics analysis based on data-independent acquisition reveals the effect of Shenling Baizhu powder (SLP) on protein expression in MAFLD rat liver tissue
Source: Clin Proteomics. 2023 Dec 1;20:55. doi: 10.1186/s12014-023-09442-9 (PMC10691125; doi:10.1186/s12014-023-09442-9)

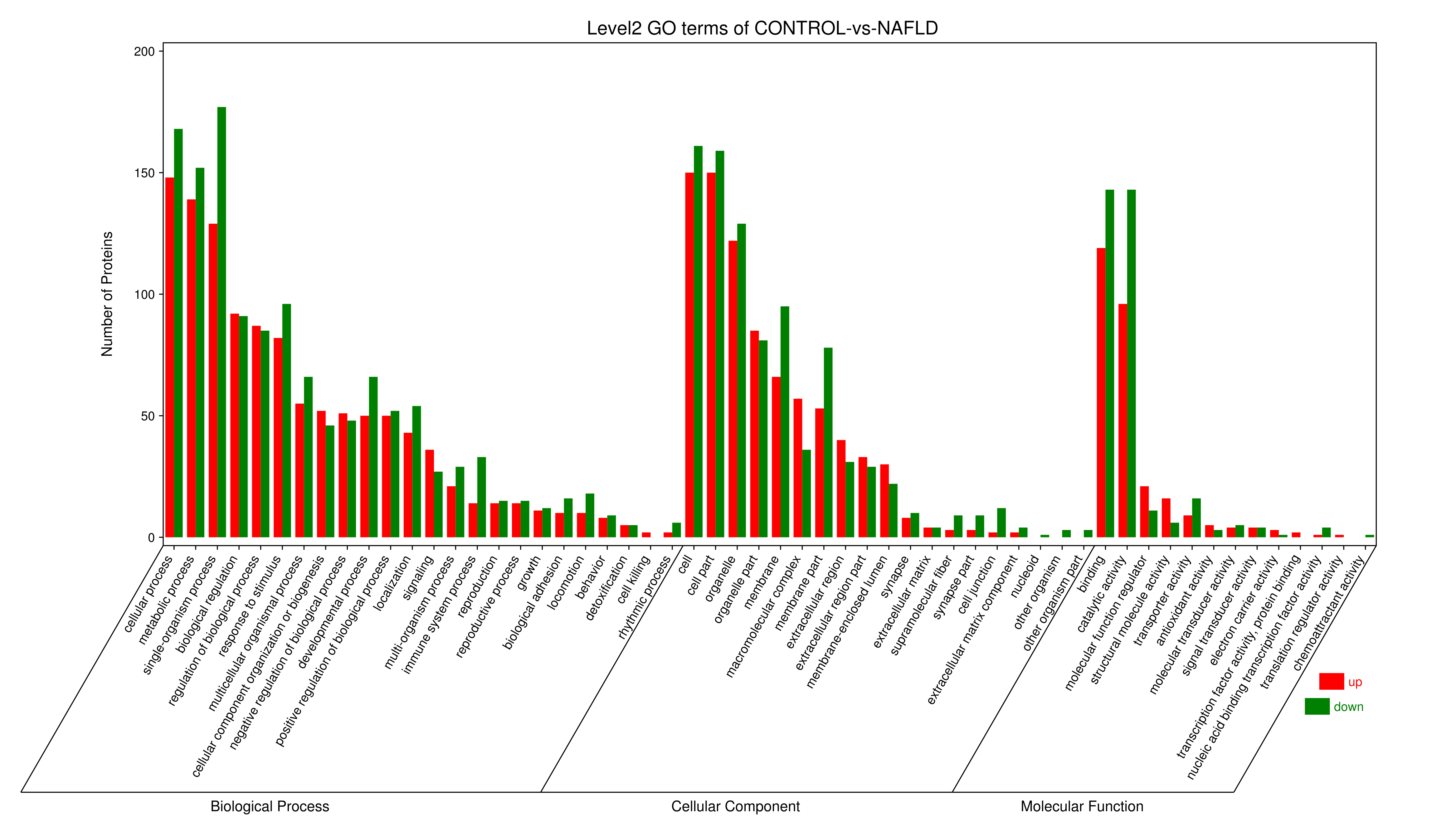

Supplement: Supplementary file 11 — Additional file 11: Figure S1. CONTROL-vs-MAFLD.GO.level2.bar. [file 12014_2023_9442_MOESM11_ESM.png]

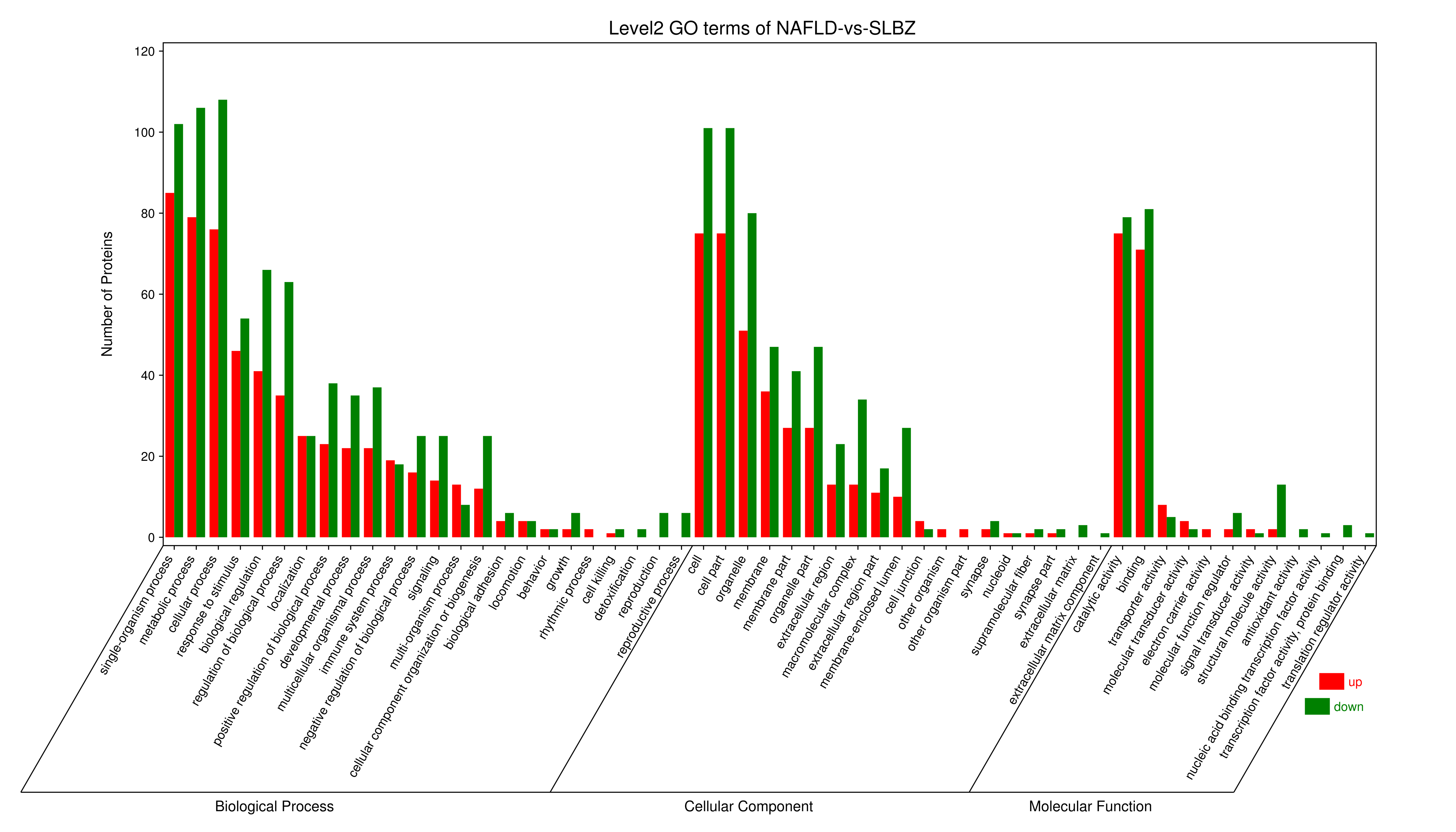

Supplement: Supplementary file 12 — Additional file 12: Figure S2. MAFLD-vs-SLBZ.GO.level2.bar. [file 12014_2023_9442_MOESM12_ESM.png]

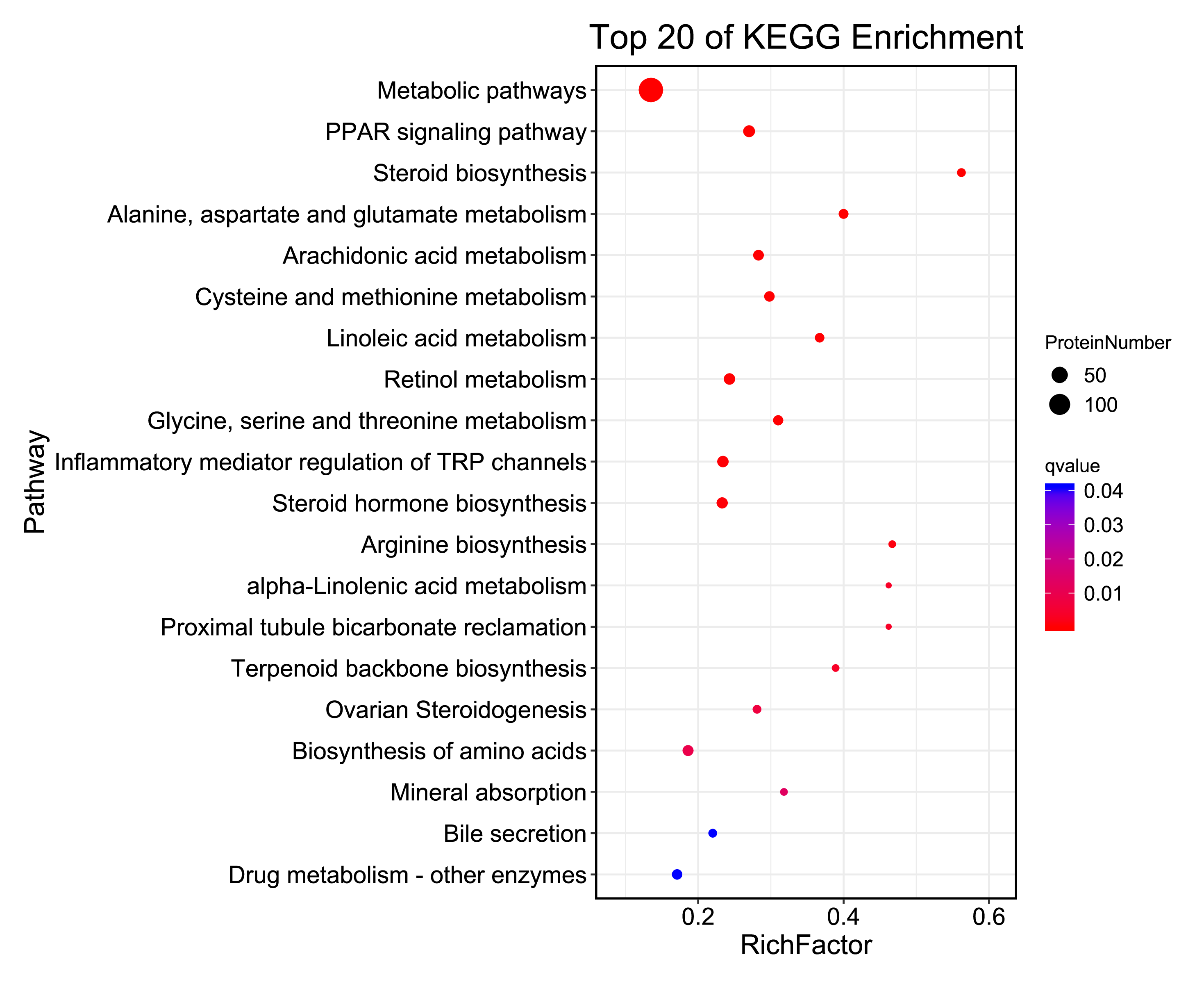

Supplement: Supplementary file 13 — Additional file 13: Figure S3. CONTROL-vs-MAFLD.gradient. [file 12014_2023_9442_MOESM13_ESM.png]

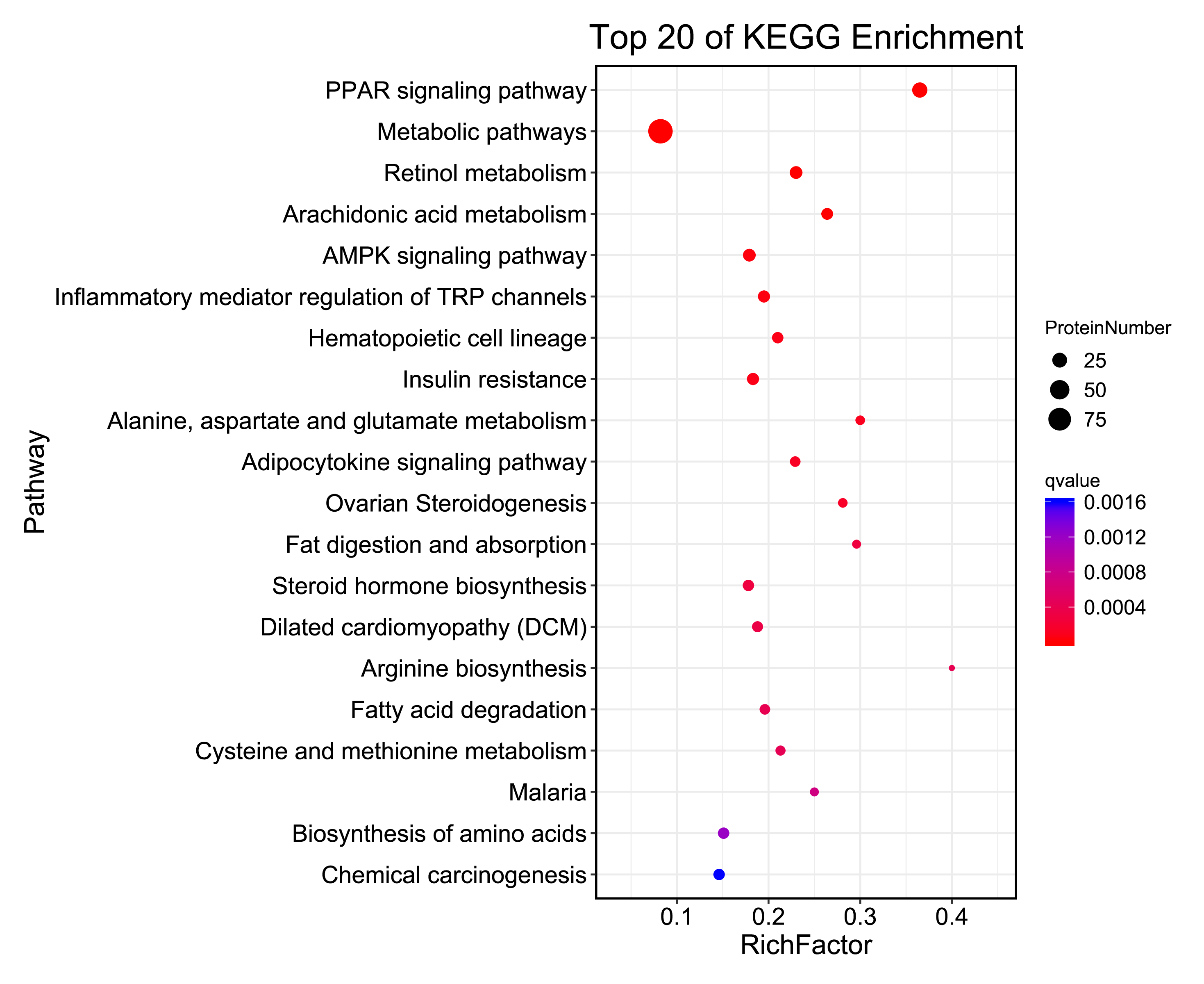

Supplement: Supplementary file 14 — Additional file 14: Figure S4. MAFLD-vs-SLBZ.gradient. [file 12014_2023_9442_MOESM14_ESM.png]
